# Lanthanum Prolongs Vase Life of Cut Tulip Flowers by Increasing Water Consumption and Concentrations of Sugars, Proteins and Chlorophylls

**DOI:** 10.1038/s41598-020-61200-1

**Published:** 2020-03-06

**Authors:** Fernando Carlos Gómez-Merino, Maribel Ramírez-Martínez, Ana María Castillo-González, Libia Iris Trejo-Téllez

**Affiliations:** 1 College of Postgraduates in Agricultural Sciences Campus Montecillo. Laboratory of Plant Nutrition. Montecillo, Texcoco, 56230 State of Mexico Mexico; 2 Chapingo Autonomous University. Institute of Horticulture. Chapingo, Texcoco, 56230 State of Mexico Mexico

**Keywords:** Plant sciences, Plant physiology

## Abstract

We evaluated the effect of separately adding two sources of lanthanum (La), LaCl_3_ and La(NO_3_)_3_ × 6H_2_O at a concentration of 40 µM each, to the preservative solution of 15 cut tulip flower varieties. Ascorbic acid (AsA; 0.2 g/L) was used as a reference solution, while distilled water was used as control. The variety Laura Fygi recorded the longest vase life with 13 days. The highest water consumption per gram of stem fresh biomass weight (FBW) (2.5 mL) was observed in the variety Violet Beauty, whereas the lowest (1.098 mL) was recorded in Pink Impression. At the end of the vase life period, higher concentrations of total soluble sugars in petals and total soluble proteins in leaves were recorded in La-treated stems, compared to the AsA treatment and the control. Additionally, La(NO_3_)_3_ × 6H_2_O supply increased the fresh weight of stems in vase and prolonged vase life. Moreover, this treatment resulted in the highest foliar concentration of chlorophylls at the end of vase life. Therefore, La increases tulip flower vase life as a consequence of improving the concentrations of some vital biomolecules.

## Introduction

Conservation of cut flowers from harvest to transport and distribution to the final consumer improves with the use of preservative solutions. Main functions of such solutions include providing sugars for energy supply, reducing proliferation of pathogenic fungi and bacteria, and preventing blockage of xylem elements in the flower stem by acidifying the medium^1^. Among preservative agents, ascorbic acid (AsA) is probably the most widely used one^2–4^, while beneficial elements such as aluminum (Al), cobalt (Co) and lanthanum (La) are emerging as novel players enhancing preservation responses in plants, fruits and flowers^5^.

Ascorbic acid serves as a co-factor for many enzymes and it contributes to the detoxification of reactive oxygen species (ROS)^3^, which renders resistance to oxidative stress and increases longevity in eukaryotic cells. For example, the application of 150 mg/L AsA significantly increased the vase life, fresh weight and percentage of total carbohydrates of snapdragon (*Antirrhinum majus*) cut flowers^6^. In gerbera (*Gerbera hybrida*) cut flowers, the application of 0.15 g/L AsA resulted in the highest anthocyanin content of petals^7^.

Lanthanum is an important rare earth element (REE) widely used in industry and medicine. In agriculture, La has shown positive effects on plant physiology and improved some yield indicators in crops when applied at low concentrations^8^ since it triggers hormesis, a dose-response phenomenon characterized by low-dose stimulation, high-dose inhibition. Hormesis may also improve cost benefit estimates for environmental contaminants, inducing beneficial/desirable effects at low doses^9^. When La is applied at low concentrations, the mean of 142% of the control is reached at 56 μM La, while the average concentration of the no-observed-adverse-effect-level (NOAEL) is 249 μM La. Importantly, factors such as intra and interspecific variations among plants tested, the pH value in the growth substrate, the concentrations of the NOAEL, and the period of time considered in the measurements may affect hormetic responses induced by La and other REEs^10–12^.

The beneficial effects of La on plants are diverse. In sweet bell pepper (*Capsicum annuum*), La improved seedling quality by enhancing some growth parameters and biomolecule concentrations, depending on the genotype and time of exposure^11^. In adzuki bean (*Vigna angularis*) seedlings, the application of lanthanum nitrate [150 mg/L La(NO_3_)_3_] improved phosphorus (P) use efficiency and tolerance to P-deficiency stress^13^. In maize (*Zea mays*) the application of 100 mg/kg La significantly increased nitrogen (N) and P in roots, as compared to the control^14^. In soybean (*Glycine max*), La increased contents of some essential nutrients, stimulated the photosynthetic rate and total chlorophyll content and led to a higher incidence of binucleated cells, resulting in a slight increase in root and shoot biomass^15^. As well, pretreatment with 20 mg/L La^3+^ in soybean alleviated the injury caused by the enhanced UV-B radiation through the regulation of the ROS production^16^. In snow lotus (*Saussurea involucrata*), the highest rooting efficiency (96%), root number/shoot (8.5), and root length (63 mm) were recorded in shoots cultured on medium containing 2.5 μM IAA combined with 100 μM La(NO_3_)_3_ × 6H_2_O^17^.

In cut flowers such as snapdragon, La (as LaCl_3_) has been shown to inhibit stem curvature of spikes by preventing several gravity-dependent processes^18^. In tulip, when La was added to the nutrient solution at concentrations less than or equal to 20 µM, foliar accumulation of essential cations such as calcium (Ca^2+^) and potassium (K^+^) was significantly increased, especially when La was supplied as LaCl_3_, as compared to La(NO_3_)_3_ × 6H_2_O^19,20^. In rose (*Rosa* × *hybrida*), the application of 500 µM/L La improved the water balance of cut flowers, increased fresh weight, reduced respiration rate, and prolonged vase life for 2–3 d more than the control^21^. Cut Easter lily (*Lilium longiflorum*) flowers treated with 60 µM LaCl_3_ underwent delayed senescence by improving antioxidant defense system and water retaining capacity^22^.

In *Arabidopsis thaliana*, La^3+^ enters the cell by clathrin-mediated endocytosis, which requires arabinogalactan proteins (AGPs) as extracellular cargo receptors in the plasma membrane^23^. In root cells, it has been shown that La^3+^ stimulates endocytosis, and the magnitude of enhancement is dependent on the dose and time of exposure to La. Such La-induced endocytosis results from DNA methylation, which is closely related to the expression level of genes encoding DNA methylases/demethylases^24^. Importantly, it was shown that both La(NO_3_)_3_ and LaCl_3_ activate endocytosis of horseradish leaf cells^25^.

The La accompanying chloride (in LaCl_3_) and nitrate [in La(NO_3_)_3_ × 6H_2_O] ions differ in the way they enter plant cells. In *Arabidopsis*, the Slowly Activating Anion Channel 1 (SLAC1) is the first characterized member of a family of anion channels (also called S-type channels) with four homologues. The channels SLAH1, SLAH2 and SLAH3 participate in NO_3_^−^ and Cl^−^ uptake and translocation to the shoot, whereas SLAC1 and SLAH3 are involved in Cl^−^ and NO_3_^−^ transport in guard cells. The SLAC/SLAH proteins are activated by different signals including carbon nutrients, dioxide fixation and water stress, in which diverse protein kinase/phosphatase complexes participate^26^.

Although a positive effect of La on postharvest quality of cut flowers has been reported, some questions remain open regarding cut tulip flowers, especially when evaluating different La sources and different tulip genotypes. For instance, what are the metabolic adjustments triggered by La during postharvest of cut tulip flowers? Do the different sources of La exert differential effects on cut tulip flower quality parameters? How are concentrations of vital molecules such as sugars, proteins and chlorophylls in cut tulip flowers changed in different tulip genotypes? To shed light on these issues, we aimed at determining the effects of separately using two La sources [LaCl_3_ and La(NO_3_)_3_ × 6H_2_O] as preservative solutions on flower quality indicators of 15 commercial tulip varieties.

## Results

### Water consumption

**Figure 1:**
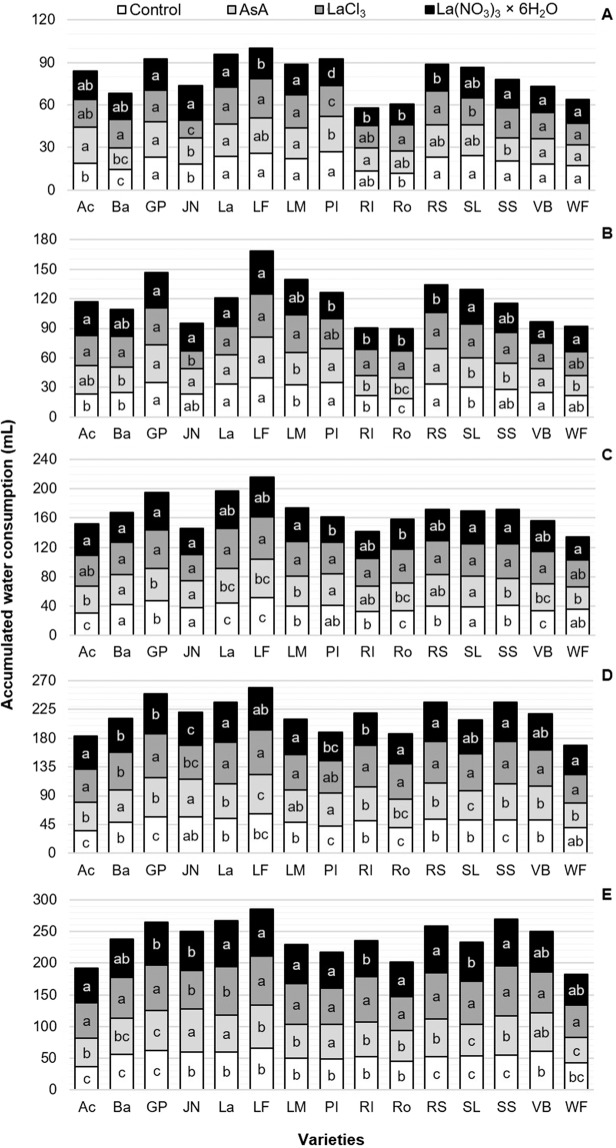
Accumulated water consumption in postharvest of evaluated cut flowers of 15 tulip varieties 3 (**A**), 5 (**B**), 7 (**C**), 9 (**D**) and 11 (**E**) days after cutting in response to different preservative solutions. Different letters in each variety (column) of each subfigure indicate statistical differences according to the Tukey test (*P* ≤ 0.05). Ac: Acropolis, Ba: Barcelona, GP: Golden Parade, JN: Jan van Nes, La: Lalibela, LF: Laura Fygi, LM: Lefeber’s Memory, PI: Pink Impression, RI: Red Impression, Ro: Rosario, RS: Red Shine, SL: Snow Lady, SS: Synaeda Show, VB: Violet Beauty, WF: World’s Favorite. Control: distilled water; AsA: L-ascorbic acid, 0.2 g/L; LaCl_3_: lanthanum(III) chloride, 40 µM; La(NO_3_)_3_ × 6H_2_O: lanthanum(III) nitrate hexahydrate, 40 µM. dac: days after cutting.

The water consumption pattern was similar in most varieties, increasing over time until reaching maximum levels during senescence (Fig. 1). However, on average, the highest consumption per day was found at 3 and 5 days after cutting (dac) (Fig. 1A,B), and it decreased after the latter date (Fig. 1C–E).

During the evaluations performed 3, 5 and 7 dac, there were 5, 3 and 2 varieties, respectively, showing no differences regarding water consumption among treatments (Fig. 1A,B). From 5 dac on, most varieties (13 of them) exhibited the highest water consumption when they were treated with preservative solution containing LaCl_3_ (Fig. 1B–E). During the evaluation carried out 11 dac, the accumulated water consumption in all varieties treated with LaCl_3_ was higher by 4.1, 14.1 and 18.8%, as compared to that observed in stems exposed to La(NO_3_)_3_, ascorbic acid and the control, respectively (Fig. 1E). The highest water consumption on average was observed in the variety Laura Fygi (72 mL), whereas the lowest was recorded in the variety World’s Favorite (46 mL), both 11 dac (Fig. 1E).

### Relative changes in fresh weight of flower stem

**Figure 2:**
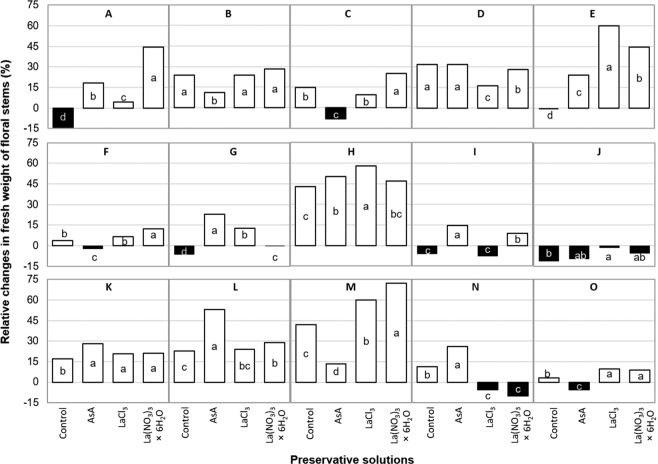
Relative changes in fresh weight of flower stems in postharvest of 15 tulip varieties evaluated 9 days after cutting in response to different preservative solutions. Different letters in each variety (subfigure) indicate statistical differences according to the Tukey test (*P* ≤ 0.05). (**A**) Ac: Acropolis, (**B**) Ba: Barcelona, (**C**) GP: Golden Parade, (**D**) JN: Jan van Nes, (**E**) La: Lalibela, (**F**) LF: Laura Fygi, (**G**) LM: Lefeber’s Memory, (**H**) PI: Pink Impression, (**I**) RI: Red Impression, (**J**) Ro: Rosario, (**K**) RS: Red Shine, (**L**) SL: Snow Lady, (**M**) SS: Synaeda Show, (**N**) VB: Violet Beauty, (**O**) WF: World’s Favorite. Control: distilled water; AsA: L-ascorbic acid, 0.2 g/L; LaCl_3_: lanthanum(III) chloride, 40 µM; La(NO_3_)_3_ × 6H_2_O: lanthanum(III) nitrate hexahydrate, 40 µM. dac: days after cutting.

Nine days after cutting, relative changes regarding fresh weight of flower stems in response to the preservative solutions were different among varieties (Fig. 2). Nonetheless, in most of them (9 out of 15 varieties), positive effects of La were observed.

Under our experimental conditions, five varieties increased flower stem weight 9 dac when they were treated with La(NO_3_)_3_ × 6H_2_O (Fig. 2A–C,F,M), whereas three of them displayed the highest increases of weight when treated with LaCl_3_ (Fig. 2E,H,O). Interestingly, in the variety Rosario no treatment increased flower stem weight 9 dac (Fig. 2J), though the lowest reduction of fresh weight was observed in stems treated with LaCl_3_. Regarding the variety Red Shine, increases in flower stem fresh weight were higher when treated with AsA, LaCl_3,_ or La(NO_3_)_3_ × 6H_2_O, as compared to the control, although no significant differences among these treatments (AsA, LaCl_3,_ or La(NO_3_)_3_ × 6H_2_O) were detected (Fig. 2K).

### Vase life

**Figure 3:**
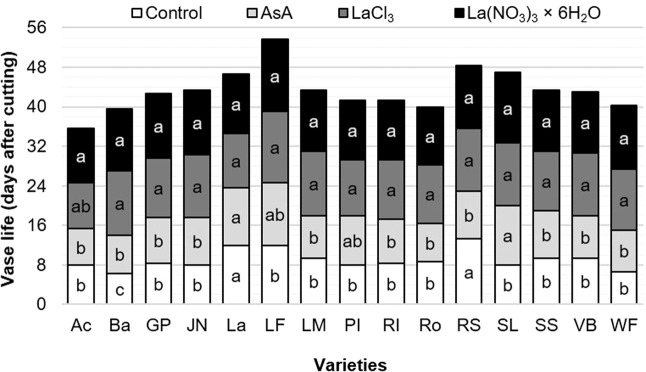
Vase life duration of flower stems in postharvest of 15 tulip varieties in response to different preservative solutions. Different letters in each column (variety) indicate statistical differences according to the Tukey test (*P* ≤ 0.05). Ac: Acropolis, Ba: Barcelona, GP: Golden Parade, JN: Jan van Nes, La: Lalibela, LF: Laura Fygi, LM: Lefeber’s Memory, PI: Pink Impression, RI: Red Impression, RS: Red Shine, Ro: Rosario, SL: Snow Lady, SS: Synaeda Show, VB: Violet Beauty, WF: World’s Favorite. Control: distilled water; AsA: L-ascorbic acid, 0.2 g/L; LaCl_3_: lanthanum(III) chloride, 40 µM; La(NO_3_)_3_ × 6H_2_O: lanthanum(III) nitrate hexahydrate, 40 µM.

On average, Laura Fygi recorded the longest vase life with 13 days, followed by Red Shine, Snow Lady, and Lalibela with 12 days. Golden Parade, Jan van Nes, Lefeber’s Memory, Synaeda Show and Violet Beauty lasted 11 days and Rosario, Pink Impression, World’s Favorite, Red Impression and Barcelona only reached 10 days. Acropolis had the shortest vase life, lasting only 8.9 days on average (Fig. 3).

The preservative solutions evaluated had significant effects on vase life. On average, in all varieties evaluated, treatments with LaCl_3_ and La(NO_3_)_3_ showed the highest number of days in vase (12.6 and 12.2 dac, respectively), as compared to the control (9 days) (Fig. 3).

### Chlorophyll concentration

At the time of cutting, the varieties with the highest leaf chlorophyll *a*, *b* and total concentrations were Rosario, Synaeda Show and World’s Favorite (Table 1). The average chlorophyll *a*, *b* and total chlorophyll concentrations were 2.84, 4.84 and 7.73 mg/g FBW, respectively. Those values decreased on the last day in vase by 7, 3 and 4%, respectively (Table 1). In these variables measured, the preservative solutions had no effect, since treatments were applied thereafter.

**Table 1:** Chlorophyll concentrations at the time of cutting of flower stem leaves of 15 tulip varieties.

Variety	Chlorophyll *a*	Chlorophyll *b*	Chlorophyll total
	mg/g FBW		
Ac	2.329 ± 0.058 fgh	3.962 ± 0.105 fgh	6.330 ± 0.127 ef
Ba	2.429 ± 0.099 fgh	4.144 ± 0.082 fgh	6.614 ± 0.166 ef
GP	2.763 ± 0.076 cdefg	4.751 ± 0.139 def	7.561 ± 0.217 de
JN	2.599 ± 0.133 defg	4.081 ± 0.171 fgh	6.723 ± 0.305 ef
La	2.565 ± 0.140 efgh	4.021 ± 0.177 fgh	6.629 ± 0.319 ef
LF	2.696 ± 0.090 cdefg	4.630 ± 0.034efg	7.371 ± 0.119 de
LM	2.171 ± 0.181 gh	3.582 ± 0.046 h	5.789 ± 0.225 f
PI	2.069 ± 0.032 h	3.765 ± 0.126 gh	5.869 ± 0.130 f
RI	3.181 ± 0.056 abcd	5.327 ± 0.188 bcde	8.562 ± 0.132 bcd
RS	2.930 ± 0.143 bcdef	5.055 ± 0.172 cde	8.034 ± 0.307 cd
Ro	3.537 ± 0.059 a	6.438 ± 0.155 a	10.035 ± 0.193 a
SL	3.272 ± 0.084 abc	5.586 ± 0.080 abcd	8.913 ± 0.163 abc
SS	3.412 ± 0.114 ab	6.118 ± 0.095 ab	9.589 ± 0.192 ab
VB	3.063 ± 0.019abcde	5.298 ± 0.162 bcde	8.413 ± 0.177 bcd
WF	3.609 ± 0.066 a	5.827 ± 0.254 abc	9.496 ± 0.314 ab

Ac: Acropolis, Ba: Barcelona, GP: Golden Parade, JN: Jan van Nes, La: Lalibela, LF: Laura Fygi, LM: Lefeber’s Memory, PI: Pink Impression, RI: Red Impression, RS: Red Shine, Ro: Rosario, SL: Snow Lady, SS: Synaeda Show, VB: Violet Beauty, WF: World’s Favorite. FBW: Fresh Biomass Weight. Different letters in each column indicate statistical differences. Data are means of three replicates, represented by a 500 mL glass jar with two flower stems. Means ± SD with different letters indicate statistical differences among treatments according to the Tukey test (*P* ≤ 0.05).

**Figure 4:**
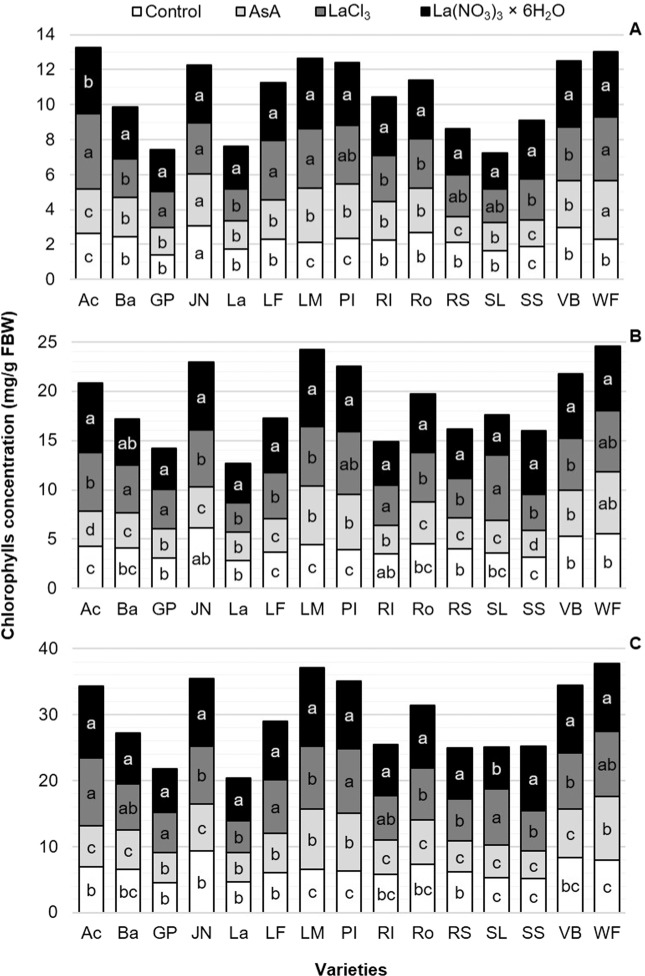
Concentration of chlorophyll *a* (**A**), *b* (**B**) and total (**C**) on the last day in the vase of stem leaves of 15 tulip varieties in postharvest in response to different preservative solutions. Different letters in each variety (column) and each subfigure indicate statistical differences according to the Tukey test (*P* ≤ 0.05). Ac: Acropolis, Ba: Barcelona, GP: Golden Parade, JN: Jan van Nes, La: Lalibela, LF: Laura Fygi, LM: Lefeber’s Memory, PI: Pink Impression, RI: Red Impression, RS: Red Shine, Ro: Rosario, SL: Snow Lady, SS: Synaeda Show, VB: Violet Beauty, WF: World’s Favorite. FBW: Fresh Biomass Weight. Control: distilled water; AsA: L-ascorbic acid, 0.2 g/L; LaCl_3_: lanthanum(III) chloride, 40 µM; La(NO_3_)_3_ × 6H_2_O: lanthanum(III) nitrate hexahydrate, 40 µM. dac: days after cutting.

In measurements carried out on the last day in vase of each variety (see Fig. 3), the concentration of chlorophyll *a* was higher with every preservative solution compared to the control in all varieties evaluated. On average, the highest concentration of chlorophyll *a* (3.21 mg/g FBW) was recorded in flower stems treated with La(NO_3_)_3_ × 6H_2_O, exceeding by 14, 38 and 41% that observed in flower stems treated with LaCl_3_, ascorbic acid and the control, respectively (Fig. 4A). On average, for chlorophyll *b* and total chlorophylls, flower stems treated with ascorbic acid showed the lowest values (5.72 and 8.98 mg/g FBW, respectively), which was statistically similar to that produced by the control with distilled water (Fig. 4B,C).

### Sugars concentration

**Figure 5:**
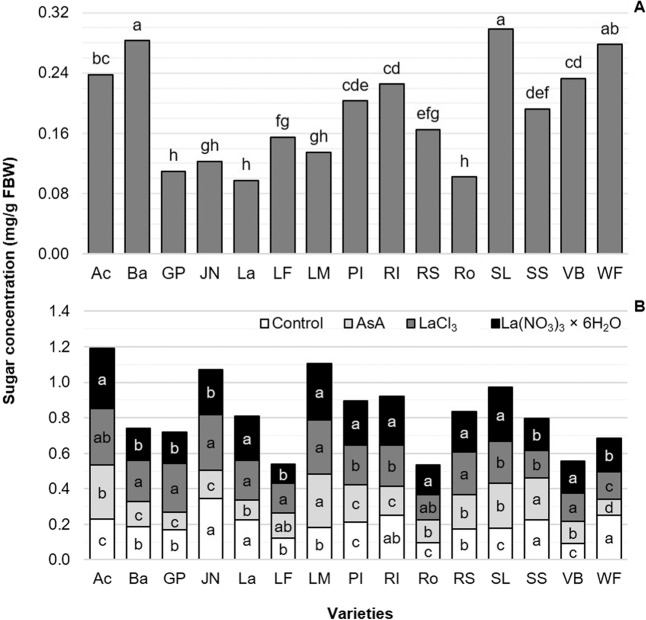
Concentration of total soluble sugars in cut flower petals of 15 tulip varieties at the time of cutting (**A**) and at the end of vase life as a function of the preservative solution evaluated (**B**). Different letters in each column (variety) of each subfigure indicate statistical differences according to the Tukey test (*P* ≤ 0.05). Ac: Acropolis, Ba: Barcelona, GP: Golden Parade, JN: Jan van Nes, La: Lalibela, LF: Laura Fygi, LM: Lefeber’s Memory, PI: Pink Impression, RI: Red Impression, RS: Red Shine, Ro: Rosario, SL: Snow Lady, SS: Synaeda Show, VB: Violet Beauty, WF: World’s Favorite. Control: distilled water; AsA: L-ascorbic acid, 0.2 g/L; LaCl_3_: lanthanum(III) chloride, 40 µM; La(NO_3_)_3_ × 6H_2_O: lanthanum(III) nitrate hexahydrate, 40 µM. FBW: Fresh Biomass Weight.

The highest concentration of sugars in petals at the time of cutting was recorded in the varieties Snow Lady, Barcelona and World’s Favorite, while the lowest sugar content at the time of cutting was found in the varieties Lalibela and Rosario (Fig. 5A). In these results, the preservative solutions had no effect, since measurements were performed before treatment applications.

At the end of the study, preservative solutions resulted in significant differences with respect to the concentration of sugars in petals. On average, the highest sugar concentration value was observed in flower stems exposed to solutions containing LaCl_3_ and La(NO_3_)_3_, with 0.225 mg/g FBW. On average, the AsA treatment produced the lowest concentration of sugars in petals, 10% below the control (Fig. 5B).

### Protein concentration

**Figure 6:**
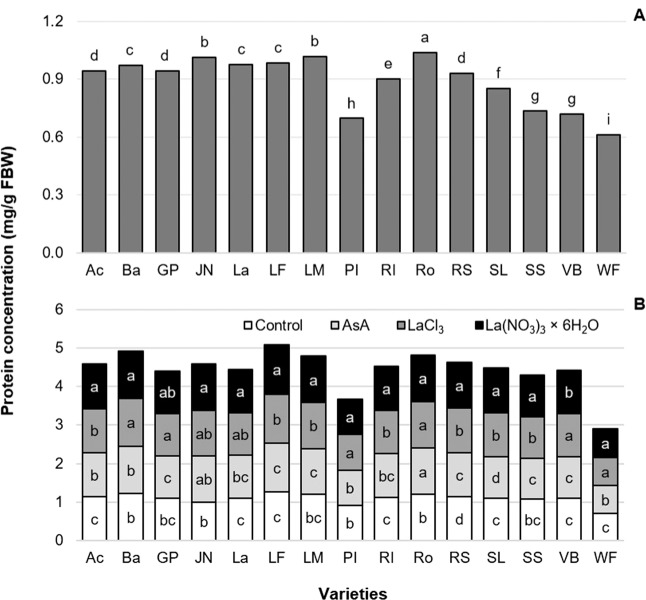
Concentration of total soluble proteins in cut flower stem leaves of 15 tulip varieties at time of cutting (**A**) and at the end of vase life as a function of the preservative solution used (**B**). Different letters in each column (variety) of each subfigure indicate statistical differences according to the Tukey test (*P* ≤ 0.05). Ac: Acropolis, Ba: Barcelona, GP: Golden Parade, JN: Jan van Nes, La: Lalibela, LF: Laura Fygi, LM: Lefeber’s Memory, PI: Pink Impression, RI: Red Impression, RS: Red Shine, Ro: Rosario, SL: Snow Lady, SS: Synaeda Show, VB: Violet Beauty, WF: World’s Favorite. Control: distilled water; AsA: L-ascorbic acid, 0.2 g/L; LaCl_3_: lanthanum(III) chloride, 40 µM; La(NO_3_)_3_ × 6H_2_O: lanthanum(III) nitrate hexahydrate, 40 µM. FBW: Fresh Biomass Weight.

Protein concentrations in leaves at the time of cutting showed significant differences among varieties. On average, protein concentration was 0.89 mg/g FBW (Fig. 6A). At the time of cutting the highest protein concentration was found in the varieties Rosario, Jan van Nes and Lefeber’s Memory, while the lowest protein concentration corresponded to the varieties Pink Impression and World’s Favorite (Fig. 6B). At this stage, the preservative solutions had no effect on the variable measured, since such treatments had not yet been applied.

At the end of the study, we observed a positive effect of supplying La with the two sources evaluated on protein concentration in leaves, while the control treatment had the lowest value (Fig. 6B).

## Discussion

In previous studies we have reported the effect of a number of La concentrations on growth, development, and nutrient concentration of tulip plants^19,20^. Moreover, we performed an in-depth analysis of the literature on La dosage resulting in beneficial effects in other plant species. For instance, in Himalayan yew (*Taxus yunnanensis*), the application of La (≤46.2 µM La^+3^ for 28 days) caused hormetic responses regarding cell growth rate and taxol production^27^. In desert broomrape (*Cistanche deserticola*), La (≤0.1 mmol/L La for 30 days) induced biomass and phenylpropanoid glycosides accumulation^28^. In horseradish (*Armoracia rusticana*), La (35 µM La^3+^) improved yield, photosynthetic rate, chlorophyll content and peroxidase activity^25^. Importantly, geometric mean and median of La concentrations inducing maximum biological response have been set at 56 and 82 µM, respectively^10^. Considering these reports and our experimental data, we decided to perform further analyses by comparing the effect of applying 0 (control) and 40 μM La on vase life, water consumption, fresh weight, and concentration of some vital biomolecules such as sugars, proteins and chlorophylls in cut flowers of 15 tulip varieties. Lanthanum was supplied as lanthanum chloride (LaCl_3_) and lanthanum nitrate hexahydrate [La(NO_3_)_3_ × 6H_2_O].

Lanthanum (La) is an element with key roles in the industry, including its catalytic and medicinal applications. In agriculture, it has been successfully applied in various crops^29,30^. Importantly, La has been proven to induce hormesis in various plant species including Himalayan yew^27^, desert broomrape^28^, horseradish^25^, eucalyptus (*Eucalyptus grandis* x *E. uroplylla*)^31^, cotton (*Gossypium hirsutum*)^32^, maize^32,33^, rice (*Oryza sativa*)^34^, common bean (*Phaseolus vulgaris*)^33^, faba bean (*Vicia faba*)^35^, soybean^15,16^, tomato (*Solanum lycopersicum*)^36^, pepper^11^, and spinach (*Spinacea oleracea*)^37,38^, among others. In ornamental plants, the effect of La has been tested in snapdragon, tulip, rose and lily^18–22^. Therefore, as compared to cereal grains and industrial crops, La has been less studied in ornamental plants or cut flowers.

Hormesis is a ubiquitous natural phenomenon of paramount importance in plant biology and agriculture nowadays. In recent reviews^9,10,12^, detailed analyses of a series of high-resolution studies have proven a substantial and significant occurrence of La-induced hormesis in plants, including stimulation of both primary and secondary metabolism.

Here, we present the stimulating effects of La on vase life and concentrations of some vital biomolecules of the primary metabolism in cut flower stems of 15 tulip varieties. Lanthanum was supplied as lanthanum chloride (LaCl_3_) or as lanthanum nitrate hexahydrate [in La(NO_3_)_3_ × 6H_2_O], which rendered differential effects on the evaluated variables. In a pioneering approach performed in barley (*Hordeum vulgare*), it was demonstrated that the rate of Cl^−^ uptake was more rapid than the rate of NO_3_^−^ uptake during the first 2 to 4 hours of treatment. Subsequently, an acceleration in the rate of NO_3_^−^ uptake after 4 hours was observed, which resulted from a more rapid, sustained uptake and transport of NO_3_^−^ providing a mobile counteranion for the cation transport, and from the synthesis of organic acids in response to NO_3_^−^ reduction, increasing the capacity for cation accumulation by providing a source of nondiffusible organic anions^39^. To date, it is well known that the ions Cl^−^ and NO_3_^−^ enter the cell using different anion channels belonging to the SLAC/SLAH family, and these channels display differential expression patterns in roots, shoots or leaves^26^. Interestingly, most hormetic dose-responses have been observed when using LaCl_3_ (55%) as compared to La(NO_3_)_3_ or La(NO_3_)_3_ × 6H_2_O^10^, which could be explained by the differential activity of the SLAC proteins involved in Cl^−^ and NO_3_^−^ uptake and transport. For instance, the rice S-type anion channel OsSLAC1 is a nitrate-selective anion channel without obvious permeability to chloride, malate, and sulfate^40^. Instead, the homolog AtSLAC1 in *Arabidopsis thaliana* has been identified as an anion channel with large permeability to both chloride and nitrate^41,42^. SLAH1, a homologue of the slow type anion channel SLAC1, modulates shoot Cl^−^ accumulation and salt tolerance in *Arabidopsis thaliana*^43^. Since plants possess diverse SLAC/SLAH channels with different tissue and cellular localizations, as well as diverse substrate selectivity^44–49^, one could expect that these families of channels could be present in the tulip genome, rendering different responses to the application of both forms of La (i.e. LaCl_3_ or La(NO_3_)_3_ × 6H_2_O). Importantly, SLAC/SLAHs play pivotal roles not only in anion uptake and transport, but also in growth, development, stress responses and phytohormone signaling^50^. However, it remains to be elucidated which intracellular regulatory elements actually control the observed hormetic dose-responses triggered by both forms of La provided under our experimental conditions. Moreover, further research will be needed to explore the tulip genome in order to identify SLAC/SLAH homologs and characterize their expression patterns and activity. Since La [either as LaCl_3_ or La(NO_3_)_3_] has been proven to enter plant cells by endocytosis^25^, the mechanisms regulating the balance between activation of SLAC/SLAH channels and endocytosis deserve further attention. In principle, La may first enter the cell by endocytosis, and once the cell detects the hormetic signal, SLAC/SLAH channels may be activated. This hypothesis coincides with the fact that under salt stress or abscisic acid (ABA), the gene activity of *SLAH1* and *SLAH3* vanished. Under control conditions SLAH1/3 heteromers together with SLAH2 release chloride and nitrate into the xylem vessels for translocation into the shoot. Upon salt stress, SLAH1 and SLAH3 expression is significantly reduced, and thus NO_3_^–^-selective SLAH2 ensures NO_3_^−^ loading of the xylem^26,51^. Under our experimental conditions, we observed more efficient effects on cut tulip flower metabolism when La was supplied as La(NO_3_)_3_ × 6H_2_O in comparison to its supply as LaCl_3_, which presumably may be attributed to the hormetic effect (i.e. eustress) produced by La.

In our study, we could observe that accumulated water consumption varied among genotypes evaluated (Table 1). The longest vase life (13 days), which occurred in Laura Fygi (Fig. 1A), was associated with greater water consumption (Table 1). It is noteworthy to mention that the vase life observed in this research is superior to that found by Benschop and De Hertogh^52^, with an average vase life of 5 days in 77 tulip varieties. Ahmed and Khurshid^53^ observed a maximum number of vase days of 9, and a minimum of 5.8 days. This behavior was associated with the genetic background of each variety.

Petal aging is generally accompanied by a loss of dry biomass weight, which is partly due to the hydrolysis of macromolecules such as sugars, proteins and nucleic acids^54^. Indeed, the longevity of the petals is directly related to their carbohydrate content. The concentrations of these molecules may remain relatively stable when flowers are attached to the plant; once flowers have been cut, the concentration of such molecules displays greater variation, since the nutrition of the petals is interrupted, and they must survive on their own reserves^55^. In our study, concentrations of all molecules measured decreased with the time course. Importantly, all molecule concentrations were higher with the application of lanthanum. In rice, the application of appropriate concentrations of La decreased the level of ROS, and hormetic effects on the antioxidant metabolism were also evident^56^. Likewise, in two marine bait algae (*Chlorella vulgaris* and *Phaeodactylum tricornutum*), the application of La(NO_3_)_3_ × 6H_2_O increased the activities of antioxidant enzymes, such as SOD and GSH^57^. Therefore, hormetic effects of La stimulating the antioxidant system can be observed in both higher and lower plants, and these effects may be responsible, at least in part, for the preservation capacity of La in cut flowers during postharvest.

Treatments tested herein differentially affected chlorophyll concentrations among varieties. According to their responses regarding chlorophyll concentrations, the evaluated varieties can be classified into three groups: (1) those in which no changes between concentrations at the time of cutting and at the end of vase life were observed (Barcelona, Laura Fygi, Violet Beauty and World’s Favorite); (2) those in which there was a decrease in chlorophyll concentrations (Golden Parade, Lalibela, Red Impression, Red Shine, Rosario, Snow Lady and Synaeda Show); and (3) those in which the chlorophyll concentrations increased (Acropolis, Jan van Nes and Pink Impression), as shown in Fig. 2. These differences may be associated with the concentrations of phytohormones in each variety. It is well documented that cytokinins and gibberellins delay the breakdown of chlorophylls, while ethylene accelerates it^58^. In plants, phytohormone biosynthesis is affected by REEs, with a concomitant effect on plant metabolism and life cycle^59^. In pineapple orchid (*Dendrobium densiflorum*), the application of the REE neodymium (5 μmol/L Nd^3+^) did not influence total levels of endogenous cytokinin but significantly increased the level of auxin^60^. In horseradish, the REE terbium (Tb^3+^) treatment decreased the auxin and gibberellic acid contents and increased the ABA content^61^. In *Arabidopsis thaliana*, the application of 10 µmol/L La alleviated ABA depression of seed germination and reversed ABA inhibition of root elongation growth^62^. Nevertheless, whether REEs are directly involved in cell signaling induced by phytohormones, and how phytohormone effects vary among species and among REEs remain as open questions.

At the end of vase life, stems treated with the preservative solution that included La(NO_3_)_3_ × 6H_2_O had the highest concentrations of chlorophylls, followed by those treated with LaCl_3_, while no statistical difference was observed between the control and the treatment with AsA (Fig. 3). Although the effect of La^3+^ in postharvest on ornamental plants has not been widely studied, in species such as spinach, maize and tobacco (*Nicotiana tabacum*), significant increases in chlorophyll content with the supply of this element have been observed, which resulted from an enhanced formation of Mg^2+^-chlorophyll or La^3+^-chlorophyll complexes^38,63,64^. In the absence of Mg^2+^, La^3+^ can replace this essential macronutrient in the chlorophyll molecule, which significantly stimulates the formation of the photosystem II (PSII) and increases the rate of transport of electrons from this photosystem^38^. In horseradish, the application of 40 µM LaCl_3_ has also stimulated chlorophyll contents^25^. In rice seedlings established in two types of soil and treated with La^3+^ (0, 30, 150, 300, 600, 900 and 1200 mg/kg LaCl_3_), the total chlorophyll concentration increased with high La doses, while the chlorophyll *a*/*b* ratio decreased by increasing the La concentration^65^. In cowpea (*Vigna unguiculata*), low La doses (0.1 to 2.5 µg/g) also increased the content of chlorophylls (*a*, *b* and total)^66^, which was also observed in pepper plants with the application of 10 µM LaCl_3_^11^ and in soybean with 0.2 mM La^3+^ ^67^.

Plant senescence is usually accompanied by an overall depletion of sugar contents. Nevertheless, such depletion does not always occur in all plant genotypes. According to van Doorn^68^, petal senescence may be caused by remobilization of sugars to other parts of the plant or an accumulation of sugars elsewhere. In many species sugar levels in petals remain high even when symptoms of senescence are already visible, as happens in some varieties of carnation (*Dianthus caryophyllus*)^69^. This phenomenon was also observed among tulip varieties in our study, and vase life duration (Fig. 1A) had no definite relationship to the concentration of sugars either at the time of cutting the stems or at the end of life vase (Fig. 4A).

Lanthanum treatments produced the highest means of vase life duration (Fig. 1B) and the concentration of sugars in petals at the end of vase life (Fig. 4B), as a function of the preservative solution. Since lanthanum can enhance photosynthesis and hence sugar biosynthesis, La treatments tested herein might have stimulated a more efficient translocation of leaf sugars to the petals^70,71^. In Chinese cabbage (*Brassica chinensis*), applications of La increased soluble sugar and vitamin C contents^72^. Similarly, a positive response to La was observed in all four pepper varieties evaluated (Sven, Sympathy, Yolo Wonder, and Zidenka) 30 dat, with increases in sugar concentrations superior to 25% as compared to the corresponding controls^11^. Likewise, increases in the contents of soluble sugars and proteins, as well as the relative water content in cut Easter lily flowers, were reported in response to the application of 60 µM La^22^.

It is well established that after cutting flower stems or after full bloom and up to senescence, there is a progressive loss of proteins^69^. However, among the tulip varieties evaluated herein, we observed increases in the foliar concentration of soluble proteins (Fig. 5A). The higher foliar protein concentration in treatments with La^3+^ (Fig. 5B) has been found to be associated with an acceleration of the transformation of inorganic N to organic forms, such as proteins^29^. Increasing the synthesis of ROS decreases the protein concentration. Since La is an element with antioxidant capacity which reduces the formation of ROS, this phenomenon in turn modifies the protein concentration^73,74^. Such antioxidant capacity of La^3+^ has been shown in cut Easter lily flowers, by increasing the activity of peroxidase, ascorbate peroxidase, glutathione reductase and glutathione peroxidase, and antioxidant metabolites like reduced ascorbic acid and reduced glutathione, while decreasing the malondialdehyde and hydrogen peroxide contents compared to the control^22^. In soybean, combined treatment with pH 4.5 acid rain and 80 µM La^3+^ promoted nitrogen assimilation synergistically^75^. Additionally, in faba (*Vicia faba*) seedlings under cadmium stress, the application of 2–120 mmol/L La^3+^ reduced the activity of proteolytic enzymes, which implied reduction of denatured proteins^76^. Importantly, in all four pepper varieties evaluated, La (10 μM LaCl_3_) stimulated soluble protein concentration 30 dat^11^. This stimulation may result from the increased uptake and translocation of nutrients such as nitrogen, thus enhancing the production of amino acids and proteins, which will act in several metabolic routes, leveraging vital plant processes^30,59^.

There have been other studies reporting the effects of La on ornamental cut flowers, including their influence on gravitropic responses^18,77^, delay of senescence, the antioxidant defense system and water retaining capacity^22^. Nonetheless, to the best of our knowledge, this is the first study reporting a detailed characterization of the metabolic and biochemical adjustments triggered by La during postharvest of cut tulip flowers, one of the top ten best-selling flowers nowadays.

## Materials and Methods

### Treatment and experimental design

In this study we used stems of 15 commercial tulip varieties from 12+ grade bulbs. Tulip bulbs were provided by the Mexican company Akiko, which is the exclusive distributor of the Dutch company Jan de Wit en Zonen B. V. (http://www.jandewitenzonen.com/en/home/) in Mexico. It is noteworthy to mention that the number 12 refers to the circumference length in cm, while the + symbol is used in commercialization to indicate bulbs which are 12 cm or more in this length.

The commercial tulip varieties used in this research were Acropolis (Ac), Barcelona (Ba), Golden Parade (GP), Jan van Nes (JN), Lalibela (La), Laura Fygi (LF), Lefeber’s Memory (LM), Pink Impression (PI), Red Impression (RI), Red Shine (RS), Rosario (Ro), Snow Lady (SL), Synaeda Show (SS), Violet Beauty (VB), and World’s Favorite (WF). Stems of all 15 commercial varieties evaluated received the same agronomic and nutritional management under greenhouse conditions. Tulip bulbs were sown individually in 2.25 L pots containing a mixture of tezontle (a local volcanic gravel; particle size 3 mm) and peatmoss at a 70:30 (v:v) ratio, respectively. For irrigation we used the Steiner nutrient solution^78^ at 50% of its original strength. All reagents used to prepare the nutrient solution were of analytic grade and the pH was adjusted to 5.5. Pots received 150 mL of the nutrient solution every other day. Once plants reached the mature stage (which depended on each variety evaluated), stems were cut at the beginning of flowering for treatment with preservative solutions.

In each tulip variety evaluated, the following vase solutions were tested: two with La, one using LaCl_3_ and the other La(NO_3_)_3_ × 6H_2_O, at a concentration of 40 µM La each; as a reference solution, L-ascorbic acid (AsA) at a concentration of 0.2 g/L was used, while distilled water was evaluated as control. Vase solutions were prepared using distilled water. Chemical sources of both AsA and La were analytical reagents provided by the company Sigma Aldrich (Darmstadt, Germany). In order to test our treatments, 15 independent assays were carried out (i.e. one assay per variety) under laboratory conditions, in an experiment with completely randomized distribution. Each variety exposed to a preservative solution had three replicates, represented by a 500 mL glass jar with two flower stems. Thus, a total of 180 experimental units were evaluated. During the carrying out of the experiment, the laboratory had mean day and night temperatures of 20 °C and 17 °C, respectively, with mean relative humidity of 40%, and 12 h light (12 μmol/m/s).

### Variables evaluated

The evaluation of response variables was done according to the phenology of each variety, which implied different time points for evaluation of each variety. In a previous study published by our research group^79^, we evaluated postharvest variables of all 15 varieties here tested, using just tap water as vase solution.

At 3, 5, 7, 9 and 11 days after cutting (dac) the flower stems, and placing them in glass jars, water consumption was measured. Glass jars contained 250 mL of the corresponding vase solution, and the volume of each glass jar was measured periodically using a 250 mL graduated cylinder.

Likewise, in each variety the relative changes (increments and losses of flower stem weight as affected by the preservative solutions) were evaluated between the day of the cut and 9 dac, by using an analytical balance (Ohaus Adventurer™ Pro; NJ, USA).

Vase life duration was assessed considering as the end of this stage (senescence phase) when the bud has between 91 and 100% silting, reduced size, petal curling and tepal thin consistency according to Azad *et al*.^80^.

Chlorophyll concentration was determined in fresh leaf tissue by the Harborne^81^ method at the time of cutting and at the end of vase life using a Genesys™ 10S spectrophotometer (ThermoFisher Scientific; Waltham, MA, USA). Absorbance was measured at 645 and 663 nm and the concentrations were estimated using the following formulas: Chlorophyll a = [(12.7*A_663_)−(2.59*A_645_)]; Chlorophyll b = [(22.9*A_645_)−(4.7*A_663_)] and Chlorophyll total = [(8.2*A_663_) + (20.2*_645_)].

The concentration of total soluble sugars in petals was measured at the time of cutting and at the end of vase life. As reference, the method described by Southgate^82^ with anthrone, sulfuric acid and 80% alcohol was used. Absorbance was determined at a wavelength of 620 nm in a Genesys™ 10S spectrophotometer. Glucose was used as standard in the preparation of the calibration curve with a concentration of between 0.1 to 1.0 mg/mL.

Protein extraction from fresh leaf tissue at the time of cutting and at the end of vase life was performed according to the method described by Höfner *et al*.^83^. Proteins were quantified using amido black solution for staining and bovine serum albumin as standard. The samples were read using a Genesys™ 10S spectrometer with an absorbance of 640 nm.

### Statistical analysis

The Shapiro-Wilk and Kolmogorov-Smirnov tests were used to verify that the data followed a normal distribution, and the Bartlett test was used to verify variance homogeneity (Supplementary Information). Data obtained in each variety were subsequently subjected to an analysis of variance and means were compared using the Tukey test (*P* ≤ 0.05), in an independent way. The Statistical Analysis System^84^ software (SAS) was used to perform all statistical analyses here presented.

## Supplementary information

Supplementary Information.
