# Supplementary material for: Lanthanum Prolongs Vase Life of Cut Tulip Flowers by Increasing Water Consumption and Concentrations of Sugars, Proteins and Chlorophylls
Source: Sci Rep. 2020 Mar 6;10:4209. doi: 10.1038/s41598-020-61200-1 (PMC7060203; doi:10.1038/s41598-020-61200-1)
Supplement: Supplementary file 1 — Supplementary Information. [file 41598_2020_61200_MOESM1_ESM.docx]

**Scientific Reports**

Supplementary Information

**Lanthanum Prolongs Vase Life of Cut Tulip Flowers by Increasing Water Consumption and Concentrations of Sugars, Proteins and Chlorophylls**

Fernando Carlos Gómez-Merino, Ana María Castillo-González, Maribel Ramírez-Martínez, and Libia I. Trejo-Téllez^*^

**Supplementary Information 1**. Kolmogorov-Smirnov and Shapiro-Wilk tests for normality and Bartlett's test for homogeneity of variance of data regarding accumulated water consumption in postharvest of evaluated cut flowers of 15 tulip (*Tulipa gesneriana* L.) varieties in response to different preservative solutions.

| **Variety** | **3 days after cutting** | | | **5 days after cutting** | | | **7 days after cutting** | | |
| --- | --- | --- | --- | --- | --- | --- | --- | --- | --- |
|  | **Pr < W** | **Pr > D** | **Pr > ChiSq** | **Pr < W** | **Pr > D** | **Pr > ChiSq** | **Pr < W** | **Pr > D** | **Pr > ChiSq** |
| **Ac** | 0.7629 | 0.1341 | 0.8258 | 0.7492 | 0.0774 | 0.6818 | 0.1967 | >0.1500 | 0.9013 |
| **Ba** | 0.2164 | 0.1369 | 0.3303 | 0.8518 | >0.1500 | 0.9713 | 0.8523 | >0.1500 | 0.8679 |
| **GP** | 0.6433 | >0.1500 | 0.9340 | 0.3231 | >0.1500 | 0.7365 | 0.2336 | >0.1500 | 0.6229 |
| **JN** | 0.8940 | >0.1500 | 0.7024 | 0.9971 | >0.1500 | 0.8308 | 0.1695 | 0.0157 | 0.5890 |
| **La** | 0.5928 | >0.1500 | 0.7024 | 0.3288 | >0.1500 | 0.2237 | 0.4962 | >0.1500 | 0.1529 |
| **LF** | 0.2072 | >0.1500 | 0.6989 | 0.9879 | >0.1500 | 0.1958 | 0.2772 | >0.1500 | 0.7046 |
| **LM** | 0.3716 | 0.0980 | 0.7015 | 0.5897 | >0.1500 | 0.3143 | 0.1717 | 0.0531 | 0.2654 |
| **PI** | 0.6422 | >0.1500 | 0.8128 | 0.3439 | 0.1198 | 0.2828 | 0.1294 | 0.0807 | 0.4747 |
| **RI** | 0.3011 | >0.1500 | 0.9681 | 0.4386 | >0.1500 | 0.9969 | 0.4998 | >0.1500 | 0.7826 |
| **RS** | 0.1017 | >0.1500 | 0.3664 | 0.4734 | >0.1500 | 0.8631 | 0.5381 | >0.1500 | 0.8202 |
| **Ro** | 0.8174 | >0.1500 | 0.3025 | 0.7186 | 0.1454 | 0.7859 | .8843 | >0.1500 | 0.8585 |
| **SL** | 0.8766 | >0.1500 | 0.9184 | 0.8626 | >0.1500 | 0.7486 | 0.0193 | 0.0667 | 0.2246 |
| **SS** | 0.1563 | >0.1500 | 0.9543 | 0.2867 | >0.1500 | 0.7859 | 0.1842 | 0.0903 | 0.2103 |
| **VB** | 0.0538 | 0.0590 | 0.6875 | 0.5171 | >0.1500 | 0.7094 | 0.6304 | >0.1500 | 0.6239 |
| **WF** | 0.0598 | 0.0650 | 1.0000 | 0.2242 | >0.1500 | 0.2273 | 0.9733 | >0.1500 | 0.7471 |

Pr < W Shapiro-Wilk test; Pr > D Kolmogorov-Smirnov test; Pr > ChiSq Bartlett test.

Ac: Acropolis, Ba: Barcelona, GP: Golden Parade, JN: Jan van Nes, La: Lalibela, LF: Laura Fygi, LM: Lefeber´s Memory, PI: Pink Impression, RI: Red Impression, RS: Red Shine, Ro: Rosario, SL: Snow Lady, SS: Synaeda Show, VB: Violet Beauty, WF: World´s Favorite.

**Supplementary Information 1** (continued).

| **Variety** | **9 days after cutting** | | | **11 days after cutting** | | |
| --- | --- | --- | --- | --- | --- | --- |
|  | **Pr < W** | **Pr > D** | **Pr > ChiSq** | **Pr < W** | **Pr > D** | **Pr > ChiSq** |
| **Ac** | 0.1057 | 0.1281 | 0.8882 | 0.1004 | 0.0914 | 0.9026 |
| **Ba** | 0.4779 | >0.1500 | 0.8679 | >0.1500 | >0.2500 | 0.4988 |
| **GP** | 0.9512 | >0.1500 | 0.5544 | 0.4413 | >0.1500 | 0.6374 |
| **JN** | 0.0740 | >0.1500 | 0.3894 | 0.1877 | >0.1500 | 0.7160 |
| **La** | 0.0892 | >0.1500 | 0.1197 | 0.0504 | >0.1500 | 0.2179 |
| **LF** | 0.3963 | >0.1500 | 0.9402 | 0.2952 | >0.1500 | 0.8412 |
| **LM** | 0.9996 | >0.1500 | 0.5063 | 0.1330 | 0.1356 | 0.5795 |
| **PI** | 0.2415 | >0.1500 | 0.4198 | 0.2840 | >0.1500 | 0.9198 |
| **RI** | 0.0592 | 0.0510 | 0.8625 | 0.1192 | >0.1500 | 0.8182 |
| **RS** | 0.4049 | >0.1500 | 0.9943 | 0.0509 | 0.0606 | 0.7033 |
| **Ro** | 0.4967 | >0.1500 | 0.6925 | 0.5515 | >0.1500 | 0.9577 |
| **SL** | 0.4450 | >0.1500 | 0.1297 | 0.3476 | >0.1500 | 0.6832 |
| **SS** | 0.7023 | >0.1500 | 0.4369 | 0.0747 | 0.1176 | 0.5916 |
| **VB** | 0.6901 | >0.1500 | 0.7033 | 0.6901 | >0.1500 | 0.8143 |
| **WF** | 0.7528 | >0.1500 | 0.3701 | 0.6822 | >0.1500 | 0.1884 |

Pr < W Shapiro-Wilk test; Pr > D Kolmogorov-Smirnov test; Pr > ChiSq Bartlett test.

Ac: Acropolis, Ba: Barcelona, GP: Golden Parade, JN: Jan van Nes, La: Lalibela, LF: Laura Fygi, LM: Lefeber´s Memory, PI: Pink Impression, RI: Red Impression, RS: Red Shine, Ro: Rosario, SL: Snow Lady, SS: Synaeda Show, VB: Violet Beauty, WF: World´s Favorite.

**Supplementary Information 2**. Kolmogorov-Smirnov and Shapiro-Wilk tests for normality and Bartlett's test for homogeneity of variance of data regarding relative changes in fresh weight of flower stems and vase life duration of flower stems in postharvest of 15 tulip (*Tulipa gesneriana* L.) varieties in response to different preservative solutions.

| **Variety** | **Relative changes in fresh weight of flower stems** | | | **Vase life** | | |
| --- | --- | --- | --- | --- | --- | --- |
|  | **Pr < W** | **Pr > D** | **Pr > ChiSq** | **Pr < W** | **Pr > D** | **Pr > ChiSq** |
| **Ac** | 0.1773 | >0.1500 | 0.7005 | 0.3631 | >0.1500 | 0.8128 |
| **Ba** | 0.0521 | 0.0705 | 0.0514 | 0.0550 | 0.0693 | 1.0000 |
| **GP** | 0.6427 | >0.1500 | 0.7912 | 0.0542 | 0.0539 | 1.0000 |
| **JN** | 0.0531 | 0.0769 | 0.9229 | 0.1277 | >0.1500 | 0.6877 |
| **La** | 0.1568 | >0.1500 | 0.0988 | 0.0592 | 0.0510 | 0.7543 |
| **LF** | 0.5687 | >0.1500 | 0.3623 | 0.1001 | >0.1500 | 0.7396 |
| **LM** | 0.1896 | >0.1500 | 0.8507 | 0.2375 | >0.1500 | 0.8336 |
| **PI** | 0.5426 | >0.1500 | 0.0539 | 0.0501 | 0.5487 | 0.9770 |
| **RI** | 0.0334 | 0.0360 | 0.9252 | 0.0821 | 0.0227 | 0.7543 |
| **RS** | 0.7789 | >0.1500 | 0.7005 | 0.0955 | 0.0802 | 0.7031 |
| **Ro** | 0.9895 | >0.1500 | 0.6075 | 0.2111 | >0.1500 | 0.7396 |
| **SL** | 0.0719 | 0.0929 | 0.5954 | 0.1840 | >0.1500 | 0.8575 |
| **SS** | 0.0722 | >0.1500 | 0.2919 | 0.0526 | 0.1500 | 0.7396 |
| **VB** | 0.1317 | 0.1193 | 0.6988 | 0.0590 | 0.0579 | 1.0000 |
| **WF** | 0.0588 | >0.1500 | 0.8341 | 0.1957 | >0.1500 | 0.5252 |

Pr < W Shapiro-Wilk test; Pr > D Kolmogorov-Smirnov test; Pr > ChiSq Bartlett test.

Ac: Acropolis, Ba: Barcelona, GP: Golden Parade, JN: Jan van Nes, La: Lalibela, LF: Laura Fygi, LM: Lefeber´s Memory, PI: Pink Impression, RI: Red Impression, RS: Red Shine, Ro: Rosario, SL: Snow Lady, SS: Synaeda Show, VB: Violet Beauty, WF: World´s Favorite.

**Supplementary Information 3**. Kolmogorov-Smirnov and Shapiro-Wilk tests for normality and Bartlett's test for homogeneity of variance of data regarding chlorophyll *a*, *b* and total in leaves of flower stems of 15 tulip (*Tulipa gesneriana* L.) varieties in response to different preservative solutions.

| **Variety** | **Chlorophyll *a*** | | | **Chlorophyll *b*** | | | **Chlorophyll total** | | |
| --- | --- | --- | --- | --- | --- | --- | --- | --- | --- |
|  | **Pr < W** | **Pr < W** | **Pr < W** | **Pr < W** | **Pr > D** | **Pr > ChiSq** | **Pr < W** | **Pr > D** | **Pr > ChiSq** |
| **Ac** | 0.0665 | 0.0521 | 0.6099 | 0.1779 | >0.1500 | 0.8067 | 0.0500 | 0.0520 | 0.2557 |
| **Ba** | 0.1242 | 0.1242 | 0.1242 | 0.5143 | >0.1500 | 0.9115 | 0.5767 | >0.1500 | 0.2083 |
| **GP** | 0.5713 | 0.5713 | 0.5713 | 0.1706 | >0.1500 | 0.7455 | 0.0909 | >0.1500 | 0.9338 |
| **JN** | 0.5900 | 0.5900 | 0.5900 | 0.2986 | >0.1500 | 0.7033 | 0.3363 | >0.1500 | 0.8846 |
| **La** | 0.0892 | 0.0892 | 0.0892 | 0.1369 | >0.1500 | 0.6577 | 0.1276 | >0.1500 | 0.3513 |
| **LF** | 0.2094 | 0.2094 | 0.2094 | 0.1258 | >0.1500 | 0.8897 | 0.1084 | 0.0797 | 0.2231 |
| **LM** | 0.3780 | 0.3780 | 0.3780 | 0.6272 | >0.1500 | 0.8705 | 0.6402 | >0.1500 | 0.8594 |
| **PI** | 0.2145 | 0.2145 | 0.2145 | 0.1212 | >0.1500 | 0.9955 | 0.0441 | >0.1500 | 0.4366 |
| **RI** | 0.1960 | 0.1960 | 0.1960 | 0.9183 | >0.1500 | 0.6464 | 0.1360 | >0.1500 | 0.2309 |
| **RS** | 0.6655 | 0.6655 | 0.6655 | 0.9988 | >0.1500 | 0.7853 | 0.8097 | >0.1500 | 0.9680 |
| **Ro** | 0.3408 | 0.3408 | 0.3408 | 0.1311 | >0.1500 | 0.7095 | 0.1689 | >0.1500 | 0.4897 |
| **SL** | 0.2693 | 0.2693 | 0.2693 | 0.0785 | 0.0584 | 0.9858 | 0.0513 | 0.0849 | 0.4197 |
| **SS** | 0.1033 | 0.1033 | 0.1033 | 0.0627 | 0.0560 | 0.4424 | 0.0691 | 0.1019 | 0.7442 |
| **VB** | 0.7110 | 0.7110 | 0.7110 | 0.2385 | >0.1500 | 0.7343 | 0.2259 | >0.1500 | 0.6281 |
| **WF** | 0.0518 | 0.0658 | 0.9814 | 0.9297 | >0.1500 | 0.9656 | 0.0570 | 0.0650 | 0.6258 |

Pr < W Shapiro-Wilk test; Pr > D Kolmogorov-Smirnov test; Pr > ChiSq Bartlett test.

Ac: Acropolis, Ba: Barcelona, GP: Golden Parade, JN: Jan van Nes, La: Lalibela, LF: Laura Fygi, LM: Lefeber´s Memory, PI: Pink Impression, RI: Red Impression, RS: Red Shine, Ro: Rosario, SL: Snow Lady, SS: Synaeda Show, VB: Violet Beauty, WF: World´s Favorite.

**Supplementary Information 4**. Kolmogorov-Smirnov and Shapiro-Wilk tests for normality and Bartlett's test for homogeneity of variance of data regarding total soluble sugar in cut flower petals and total proteins in cut flower stem leaves of 15 tulip (*Tulipa gesneriana* L.) varieties in response to different preservative solutions.

| **Variety** | **Total soluble sugars in cut flower petals on the last day in the vase** | | | **Total proteins in cut flower stem leaves on the last day in the vase** | | |
| --- | --- | --- | --- | --- | --- | --- |
|  | **Pr < W** | **Pr > D** | **Pr > ChiSq** | **Pr < W** | **Pr > D** | **Pr > ChiSq** |
| **Ac** | 0.0639 | 0.0438 | 0.4412 | 0.7426 | >0.1500 | 0.9510 |
| **Ba** | 0.7753 | >0.1500 | 0.9456 | 0.1336 | >0.1500 | 0.9068 |
| **GP** | 0.2925 | >0.1500 | 0.7089 | 0.7917 | >0.1500 | 0.8866 |
| **JN** | 0.2522 | >0.1500 | 0.3476 | 0.0501 | 0.0700 | 0.0613 |
| **La** | 0.0567 | 0.1120 | 0.8161 | 0.9973 | >0.1500 | 0.4941 |
| **LF** | 0.6656 | >0.1500 | 0.9247 | 0.5681 | >0.1500 | 0.5901 |
| **LM** | 0.0612 | 0.0503 | 0.1180 | 0.5741 | >0.1500 | 0.9228 |
| **PI** | 0.1252 | >0.1500 | 0.6243 | 0.1266 | >0.1500 | 0.7637 |
| **RI** | 0.1594 | >0.1500 | 0.9416 | 0.7270 | >0.1500 | 0.8329 |
| **RS** | 0.5042 | 0.1484 | 0.7318 | 0.7740 | >0.1500 | 0.5601 |
| **Ro** | 0.4612 | >0.1500 | 0.8906 | 0.8262 | >0.1500 | 0.8527 |
| **SL** | 0.5846 | >0.1500 | 0.4145 | 0.0524 | 0.0684 | 0.7595 |
| **SS** | 0.2972 | >0.1500 | 0.5654 | 0.4526 | >0.1500 | 0.9232 |
| **VB** | 0.4951 | >0.1500 | 0.4300 | 0.2661 | >0.1500 | 0.6173 |
| **WF** | 0.8180 | >0.1500 | 0.7503 | 0.0587 | 0.0811 | 0.9942 |

Pr < W Shapiro-Wilk test; Pr > D Kolmogorov-Smirnov test; Pr > ChiSq Bartlett test.

Ac: Acropolis, Ba: Barcelona, GP: Golden Parade, JN: Jan van Nes, La: Lalibela, LF: Laura Fygi, LM: Lefeber´s Memory, PI: Pink Impression, RI: Red Impression, RS: Red Shine, Ro: Rosario, SL: Snow Lady, SS: Synaeda Show, VB: Violet Beauty, WF: World´s Favorite.
